# Calciprotein particles disrupt autophagy in vascular endothelial cells and smooth muscle cells

**DOI:** 10.1016/j.athplu.2026.100558

**Published:** 2026-03-20

**Authors:** Negar Sharifimoghaddammood, Isabel Pintelon, Winnok H. De Vos, Lynn Roth, Cédric H.G. Neutel

**Affiliations:** a Laboratory of Physiopharmacology, University of Antwerp, Antwerp, Belgium; b Laboratory of Cell Biology and Histology, University of Antwerp, Antwerp, Belgium; c Antwerp Centre for Advanced Microscopy (ACAM), University of Antwerp, Universiteitsplein 1, 2610, Antwerp, Belgium; d μNEURO Research Excellence Consortium On Multimodal Neuromics, University of Antwerp, Universiteitsplein 1, 2610, Antwerp, Belgium; e Department of Biomedical Engineering, CARIM School for Cardiovascular Diseases, Maastricht University, the Netherlands

**Keywords:** Calciprotein particles, Autophagy, VSMC, Endothelium

## Abstract

Calciprotein particles (CPPs) act as buffers against mineral overload, but during prolonged mineral stress they convert from the small, amorphous CPP1 form into large, crystalline CPP2. This shift is associated with endothelial dysfunction and arterial stiffness. In this brief report, we assessed the impact of CPPs on autophagy, a key intracellular homeostatic process for maintaining (cardio)vascular health, in human aortic endothelial cells (HAoECs), human aortic smooth muscle cells (HAoSMCs), and murine eGFP-mRFP-LC3 vascular smooth muscle cells (VSMCs). In HAoECs, both CPP1 and CPP2 (100 μg/mL [Ca^2+^], 24 h) increased LC3-II levels and reduced autophagic flux. In contrast, HAoSMCs showed impaired flux only after exposure to CPP2. In murine VSMCs, CPP2 reduced autolysosome formation without affecting autophagosome numbers. Together, these findings indicate that CPPs interfere with lysosomal function and autophagic flux, with CPP2 exerting a stronger effect in smooth muscle cells. This supports a mechanistic link between chronic mineral stress and vascular disease and suggests that enhancing autophagy could potentially help counteract CPP-associated cardiovascular risk.

## 1 Introduction

Calciprotein particles (CPPs) are nanoscale protein–mineral complexes that naturally form in biological fluids when calcium and phosphate concentrations become supersaturated in the presence of proteins. The formation of CPPs in the circulation functions to prevent crystallization due to surges of Ca-P_i_ (*i.e.,* mineral stress) and are cleared rapidly from the bloodstream [1]. However, in conditions of persistent mineral stress, such as in chronic kidney disease the level of circulating CPPs increases [2]. Moreover, prolonged oversaturation with mineral ions creates progressively larger CPPs, pushing the transition of small amorphous CPPs (primary CPP/“CPP1”) towards larger, crystalline particles (secondary CPP/“CPP2”) [1]. As CPPs become too big to be efficiently cleared by the kidneys, they are cleared from the circulation by endothelial cells and macrophages [3,4]. Previous studies have demonstrated that the uptake of CPPs in the larger arteries induces endothelial cell dysfunction and increases arterial stiffness, a strong independent risk factor for cardiovascular disease [4,5]. However, the underlying mechanisms remain elusive. This *in vitro* study evaluated the effect of CPPs on vascular cell autophagy, a key intracellular homeostatic process that is essential for maintaining vascular health [6]. Autophagy acts as a protective mechanism by degrading damaged organelles and proteins, thereby preventing the accumulation of toxic cellular debris that can trigger fibrosis, inflammation, and cardiovascular disease [7,8]. By focusing on autophagy, we aim to elucidate how CPP-induced disruption of this process may contribute to vascular dysfunction.

## 2 Methods

### 2.1 Synthesis and characterization of calciprotein particles (CPPs)

Calciprotein particles (CPPs) were generated *in vitro* in pre-warmed DMEM (w/o phenol red), supplemented with 10% heat-inactivated FBS. Phosphate (NaH_2_PO_4_/Na_2_HPO_4_,19%/81% mixture, 1M) and calcium (CaCl_2_,1M) stock solutions were used to increase [PO_4_^3−^] and [Ca^2+^] in the DMEM/FBS solution to 3.5 mM and 1 mM, respectively. Phosphate was added first, followed by vortexing, after which calcium was added, followed again by vortexing. The solution was incubated at 37 °C for either 1 day or 7 days to generate primary (CPP1) or secondary (CPP2) CPPs, respectively. The CPP-containing solution was centrifuged at.

20,000×*g* for 90 min. The CPP-containing pellet was resuspended in sterile phosphate-buffered saline (PBS) to generate a “CPP batch”. CPP batches were freshly prepared for every individual experiment, since freeze-thaw cycles would affect CPP stability and function. In addition, CPPs were visualized with transmission electron microscopy (TEM). Briefly, CPPs were diluted 1:10 in MilliQ water after which a sample (1 μL) was applied on Formvar-coated nickel grids. The samples were dried at room temperature after which they were imaged via a Tecnai G2 Spirit Bio TWIN microscope (Thermo Fisher Scientific, Eindhoven, The Netherlands) at 120 kV, without staining. Calcium content of the CPPs was measured by flame atomic absorption spectrometry (FAAS). A concentration of 100 μg/mL [Ca^2+^] was used as a working concentration for CPPs *in vitro*, based on previously published studies and prior experiments within our research group, in which this dose has been widely applied. This concentration falls within the pathophysiological relevant range reported for calcified tissues, such as atherosclerotic plaques, and enables direct comparison of our findings with existing CPP literature as well as with our own previous work [1,3,5,9,10].

### 2.2 Measuring autophagic flux *in vitro*

Autophagy is a dynamic process that begins with the formation of autophagosomes, which sequester intracellular cargo such as damaged organelles and misfolded proteins. The autophagosomes then fuse with lysosomes, resulting in the degradation of their contents. The term “autophagic flux” describes the complete progression of this pathway over time and is typically assessed by monitoring LC3-II turnover via western blotting, both in the presence and absence of lysosomal degradation inhibition, commonly achieved using Bafilomycin A1, a V-ATPase inhibitor [11]. To this end, human aortic endothelial cells (HAoECs, Merck Life Sciences, 304-05A) and human aortic vascular smooth muscle cells (HAoSMCs, Merck Life Sciences, 354-05A) were treated for 24 h with either CPP1 or CPP2 (100 μg/mL [Ca^2+^]) in either the presence or absence of bafilomycin A1 (Santa Cruz Biotechnology, sc-2021550). PBS was used as control to the CPPs. 160 nM of bafilomycin A1 was added during the last 3 h of the treatment period, after which the cells were lysed and western blotting for LC3-II was performed. Autophagic flux for each treatment group was calculated as the relative difference in LC3-II signal between the bafilomycin untreated and pre-treated samples, thereby reflecting the net autophagic activity due to lysosomal inhibition within each experimental condition. Additionally, murine primary aortic VSMCs were isolated from mRFP-EGFP-LC3 transgenic C57Bl6/J mice. In brief, the aorta was cleaned from perivascular adipose tissue and digested using a combination of elastase and collagenase. A second incubation with enzymes was performed after having stripped the adventitial layer from the aorta. Isolated cells were centrifuged and resuspended in fresh cell culture medium. Cells were used between passage 4 and 10. Primary VSMCs were treated for 24 h with either CPP1 or CPP2 (100 μg/mL [Ca^2+^]), after which fluorescent images of formaldehyde-fixated plates were acquired by an automated Nikon Ti-E inverted microscope (Nikon Instruments Europe, Amsterdam, The Netherlands). The microscope was equipped with a SPECTRA light engine® solid-state light source (Lumencor, Beaverton, USA) and a Nikon CS-Ri2 digital camera. Image acquisition was performed with Nikon NIS-elements software. 16 images were taken per well using the following excitation band pass filters: 395 nm (DAPI; Cell nucleus), 470 nm (GFP), 555 nm (CY3/RFP), and 640 nm (CY5; HCS Cellmask™). HCS Cellmask™ (Thermo Fisher Scientific, H32721) was used for visualizing cell cytoplasm. A 20× dry objective (numerical aperture 0.75) was used for acquiring images. Separation of different fluorescence channels was done using quadruple dichroic mirrors and the following emission band pass filters: 422-448 nm (DAPI), 470-530 nm (EGFP), 565-605 nm (CY3/RFP), 669-741 nm (CY5/HCS Cellmask). Image processing was performed in Fiji. Quantification of fluorescent LC3 dots was done using “CellBlocks.ijm”, a script for automated cell-based analysis for FIJI freeware. In brief, the script segments nuclei (using DAPI), cells (using HCS Cellmask, H32721, ThermoFisher Scientific) and intracellular spots (LC3 fluorescent spots). Only spots located within the cytoplasm were retained for further analysis. The number of spots per cell was extracted from each image and averaged per well. Spots overlapping by more than one pixel in both the RFP and GFP channels were classified as positive for both markers, indicating autophagosomes (yellow dots). For quantification, only cells containing at least one autophagosome were included. Initially, imaging targeted approximately 100 cells per well; following quality control and exclusion of unsuitable images, the number of analysed cells per well ranged from 50 to 250, reflecting variability in image quality and cell detection. Spot counts were measured on a per-cell basis, averaged first per well, and then across three technical replicate wells per treatment. Each treatment was performed in three independent biological replicates.

### 2.3 Western blotting

HAoECs and HAoSMCs were collected in Laemmli sample buffer (Bio-Rad), supplemented with β-mercaptoethanol (5%), to facilitate cell lysis. Samples were heat-denatured for 5 min at 100 °C. Afterwards, samples were loaded on Bolt 4-12% bis-tris gels (Invitrogen) for gel electrophoresis, followed by wet transfer on polyvinylidene fluoride membranes. Membranes were blocked in Odyssey® Blocking Buffer (Li-Cor Bioscience) and probed with primary antibody (overnight, 4 °C). Following primary antibodies and dilutions were used: anti-β-actin (1/2000 dilution, Abcam; 8226), anti-LC3 (1/1000 dilution, Nanotools, 0231-100/LC3-5F10). Subsequently, (IR)-conjugated secondary antibodies (anti-rabbit: IgG926-32211 and anti-mouse: IgG926-68070; Li-Cor Biosciences) were used for IR fluorescence detection using an Odyssey SA infrared imaging system (LI-COR Biosciences). Western blot signal was analysed using Image Studio Lite (LI-COR).

### 2.4 Statistics

First, data distribution was assessed using the Shapiro–Wilk normality test. As all data passed normality testing, data are presented as mean ± SD, with “n” indicating the number of independent biological replicates, as indicated in the figure legends. Statistical analyses were performed using GraphPad Prism 10.0 (GraphPad Software, La Jolla, CA, USA). Specific statistical tests are detailed in the figure legends.

## 3 Results

**Fig. 1:**
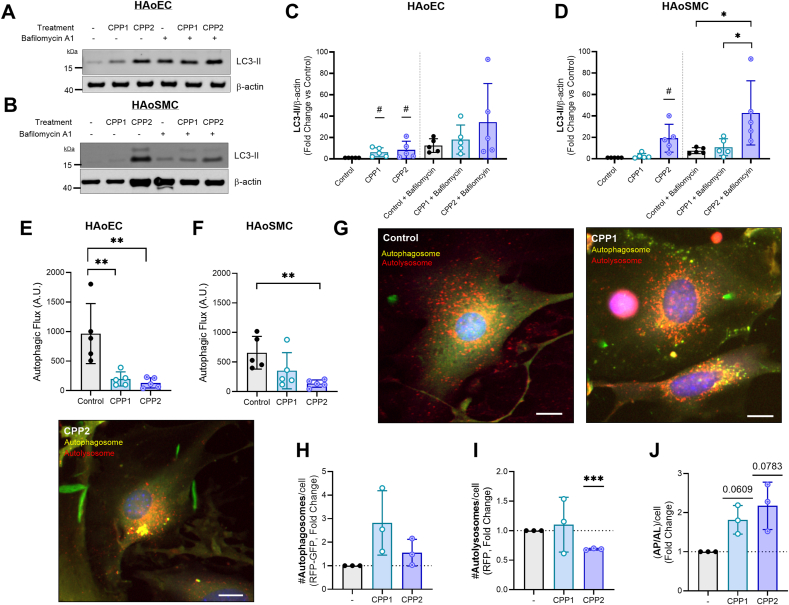
**Calciprotein particles disrupt LC3-mediated autophagic flux in vascular cells.** Human aortic endothelial cells (HAoECs) and human aortic smooth muscle cells (HAoSMCs) were treated for 24 h with CPP1 or CPP2. **(A, B)** A series of immunoblots were performed to investigate LC3-mediated autophagic activity. **(C, D)** LC3-II levels in both HAoECs and HAoSMCs after CPP treatment. **(E, F)** Autophagic flux in both HAoECs and HAoSMCs after CPP treatment. **(G)** Using primary RFP-GFP-LC3 murine aortic VSMCs, autophagic flux was analysed in a western blot-independent manner. Scale bar = 20 μm**. (H**–**J)** Analysis of RFP(-GFP) signal after CPP incubation. Yellow dots represent autophagosomes (RFP-GFP) and red dots represent autolysosomes (RFP). Statistical analyses: (C,D) one-sample *t*-test(s) (on Log_2_ transformed data, hypothetical value = 0) were used to compare against control. #p < 0.05. A One-Way ANOVA was used to compare between treatment group (excluding comparison against control) with a Tukey post hoc test for multiple comparisons. n = 5. ∗p < 0.05 (E, F) One-way ANOVA with a Dunnett's test for multiple comparisons. n = 5. (H-J) One-sample *t*-test(s) on Log_2_ transformed data, hypothetical value = 0. n = 3. ∗p < 0.05, ∗∗p < 0.01, ∗∗∗p < 0.001. CPP1 = primary calciprotein particle; CPP2 = secondary calciprotein particle; AP/AL = Autophagosome/Autolysosome.

Human aortic endothelial cells (HAoECs), treated with CPP1 and CPP2 (both in a concentration of 100 μg/mL [Ca^2+^]) for 24 h, revealed a significant increase of LC3-II levels in the absence of 160 nM bafilomycin (Fig. 1A–C). There were no significant differences in LC3-II levels between the groups after additional bafilomycin A1 treatment. The autophagic flux, determined as the relative difference between the samples with and without bafilomycin A1, revealed a significantly decreased autophagic flux in HAoECs after 24 h incubation with both CPP1 and CPP2 (Fig. 1E). Interestingly, LC3-II levels in human aortic smooth muscle cells (HAoSMCs) were significantly increased after CPP2 treatment, but not with CPP1, in both the absence and presence of bafilomycin A1 (Fig. 1B–D). Furthermore, only CPP2 caused a significant decrease in autophagic flux in HAoSMCs (Fig. 1F).

Further, primary murine vascular smooth muscle cells (VSMCs), isolated from eGFP-mRFP-LC3 reporter mice, were used to expand on the abovementioned findings in HAoSMCs. eGFP-mRFP-LC3 VSMCs were incubated for 24 h with CPP1 or CPP2 (Fig. 1G). Interestingly, whereas no significant differences were found in the number of autophagosomes per cell, identified as the number of yellow dots, there was a significant decrease in the number of autolysosomes per cell, identified as red dots, after CPP2 treatment (Fig. 1H and I). Furthermore, while an increase was observed in the autophagosome/autolysosome ratio (“AP/AL”), this was not significant after statistical analysis (Fig. 1J).

## 4 Discussion

The uptake of calciprotein particles (CPPs) in vascular endothelial cells and vascular smooth muscle cells has been suggested to play a role in endothelial cell (EC) dysfunction, smooth muscle cell (SMC) calcification, and arterial stiffening [2,4,5]. However, the underlying mechanisms remain unclear and clarifying them will be crucial in both understanding CPP-related cardiovascular disease as well as to identify potential treatment targets. This study demonstrated that CPPs inhibit LC3-mediated autophagic activity in both endothelial cells (ECs) and vascular smooth muscle cells (SMCs) *in vitro*, with CPP2 suppressing autophagy in both cell types while CPP1 inhibited autophagy exclusively in ECs. These results are in line with earlier *in vitro* reports where lysosomal dysfunction due to CPPs was found to induce blockage of autophagic flux in renal proximal tubule cells [12,13]. The intracellular accumulation of CPPs and their high calcium and phosphate content decrease lysosomal function thereby inhibiting autophagic flux [12]. This is detrimental, as studies have shown that decreased autophagic activity is linked to decreased cardiovascular function and a higher incidence of cardiovascular disease [6]. Fortunately, autophagy can be induced both pharmacologically and through lifestyle changes (e.g., caloric restriction) [6]. This makes that the induction of autophagic flux has become an active and widely explored approach across many research areas, including cardiovascular disease [6,14]. Further research should therefore investigate whether the detrimental effects of CPPs on vascular cells and cardiovascular function (e.g., arterial stiffness) can be attenuated by inducing autophagy and/or promoting lysosomal function.

## Declaration of competing interest

The authors declare that they have no known competing financial interests or personal relationships that could have appeared to influence the work reported in this paper.
